# Brain radiation injury leads to a dose- and time-dependent recruitment of peripheral myeloid cells that depends on CCR2 signaling

**DOI:** 10.1186/s12974-016-0496-8

**Published:** 2016-02-03

**Authors:** Michael J. Moravan, John A. Olschowka, Jacqueline P. Williams, M. Kerry O’Banion

**Affiliations:** Department of Radiation Oncology, University of Rochester School of Medicine and Dentistry, Rochester, NY USA; Department of Neuroscience, University of Rochester School of Medicine and Dentistry, Rochester, NY USA; Department of Environmental Medicine, University of Rochester School of Medicine and Dentistry, Rochester, NY USA; Department of Neurology, University of Rochester School of Medicine and Dentistry, Rochester, NY USA

**Keywords:** Ionizing radiation, Late CNS radiation injury, Chimeric mice, Cell infiltration, Myeloid cells, CCR2

## Abstract

**Background:**

Cranial radiotherapy is used to treat tumors of the central nervous system (CNS), as well as non-neoplastic conditions such as arterio-venous malformations; however, its use is limited by the tolerance of adjacent normal CNS tissue, which can lead to devastating long-term sequelae for patients. Despite decades of research, the underlying mechanisms by which radiation induces CNS tissue injury remain unclear. Neuroinflammation and immune cell infiltration are a recognized component of the CNS radiation response; however, the extent and mechanisms by which bone marrow-derived (BMD) immune cells participate in late radiation injury is unknown. Thus, we set out to better characterize the response and tested the hypothesis that C-C chemokine receptor type 2 (CCR2) signaling was required for myeloid cell recruitment following brain irradiation.

**Methods:**

We used young adult C57BL/6 male bone marrow chimeric mice created with donor mice that constitutively express enhanced green fluorescent protein (eGFP). The head was shielded to avoid brain radiation exposure during chimera construction. Radiation dose and time response studies were conducted in wild-type chimeras, and additional experiments were performed with chimeras created using donor marrow from CCR2 deficient, eGFP-expressing mice. Infiltrating eGFP+ cells were identified and quantified using immunofluorescent microscopy.

**Results:**

Brain irradiation resulted in a dose- and time-dependent infiltration of BMD immune cells (predominately myeloid) that began at 1 month and persisted until 6 months following ≥15 Gy brain irradiation. Infiltration was limited to areas that were directly exposed to radiation. CCR2 signaling loss resulted in decreased numbers of infiltrating cells at 6 months that appeared to be restricted to cells also expressing major histocompatibility complex class II molecules.

**Conclusions:**

The potential roles played by infiltrating immune cells are of current importance due to increasing interest in immunotherapeutic approaches for cancer treatment and a growing clinical interest in survivorship and quality of life issues. Our findings demonstrate that injury from brain radiation facilitates a dose- and time-dependent recruitment of BMD cells that persists for at least 6 months and, in the case of myeloid cells, is dependent on CCR2 signaling.

**Electronic supplementary material:**

The online version of this article (doi:10.1186/s12974-016-0496-8) contains supplementary material, which is available to authorized users.

## Background

Cranial radiotherapy is frequently utilized as part of the treatment of primary and secondary brain tumors, prophylaxis against clinically occult systemic malignancies, and for non-neoplastic diseases, such as arterio-venous malformations. However, the use of curative doses of radiation is often limited due to the potential for damage in the adjacent and surrounding normal brain tissue. Indeed, both acute and late/delayed radiation-induced sequelae can result in significant morbidity and/or mortality for patients, and thus, the pathogenesis of radiation injury in the normal central nervous system (CNS) tissue has been the subject of investigation for decades. Unfortunately, a thorough understanding of the underlying mechanisms, leading to mitigation strategies, has remained elusive.

Although identified as a frequent constituent of acute and late effects seen following radiation exposure, the precise contribution of neuroinflammation to their initiation and progression in the brain is not entirely clear. Work from our group [1–3] and others [4–6] has suggested a critical role for inflammation in the normal CNS response to radiation. Furthermore, use of anti-inflammatory therapies, such as post-therapy steroids and NSAIDs, has demonstrated some beneficial outcomes, both in preclinical studies [7–9] and in patients [10–12]. However, the complex interplay between the various components of the inflammatory and immune systems has limited the ability of researchers to identify more specific targets that could effectively mitigate CNS normal tissue effects. For example, our group previously demonstrated acute and persistent increases in the numbers of CD3+ cells and CD11c+ cells in the CNS, as well as increased expression of MHC II, in response to radiation [3]. However, the development of specific and targeted therapeutic approaches would require identification of the origin of these CD11c+ and MHC II+ cells, since both populations could originate either from endogenous microglia or from leukocytes that have infiltrated from the periphery.

Multiple laboratories have investigated leukocyte infiltration in the CNS and have shown that bone marrow-derived cells have the ability to enter and persist within the CNS, with a tremendous potential for transformation [13–22]. Importantly, the rate of leukocyte infiltration into the CNS has been shown to increase in the setting of inflammation and injury [21, 23–27], and an intriguing study, examining the infiltration of peripherally derived immune cells using bone marrow transplanted animals, suggested that leukocytes recruited to the CNS can develop a dendritic cell phenotype [17], although there is debate as to whether dendritic cells could also arise from resident microglia [28–30]. Burrell et al. showed that cranial irradiation led to a time- and dose-dependent infiltration of bone marrow-derived (BMD) cells, specifically in areas directly exposed to the radiation beam, starting at 7 days and persisting for up to 1 month post-irradiation. About 50 % of the BMD cells located in the brain parenchyma stained for the microglial marker Iba-1, 7 days following radiation [31]. Only a very small number of the BMD cells were found to express smooth muscle cell markers, and none were found to express endothelial or other glial markers. It is unclear whether these cells persisted later than 1 month post-radiation or if they contribute to the CD11c+ cells we previously reported [3].

Recruitment of myeloid lineage cells into the injured CNS environment depends on the expression of endothelial activation markers, such as intracellular adhesion molecule-1 (ICAM-1) [32–34] and chemokine signaling, particularly through the C-C (motif) ligand-2 (CCL2)/C-C chemokine receptor type 2 (CCR2) [35–37] and stromal cell-derived factor (SDF1)/CXCR4 pathways [38, 39]. The ICAM-1 and CCL2/CCR2 pathways have also been shown to play a role in the recruitment of dendritic cells into injured/inflamed tissues, including the brain [35, 40–43]. Significantly, previous studies from our laboratory have demonstrated increased messenger RNA (mRNA) levels of CCL2 and ICAM-1 following CNS radiation exposure at times as late as 6–12 months post-irradiation [3, 33]. A recent study by Morganti et al. demonstrated an increase in the number of CD11b+/F4-80+/CX3CL1-/CCR2+ cells (assumed to be peripherally derived), a significant increase in the CCL2 protein level, and an increase in the ICAM-1 mRNA level in the brain at 7 days following 10 Gy of cranial irradiation, suggesting that CCR2 signaling is responsible for the recruitment of these cells [44]. Taking all these findings together, we have hypothesized that the time-dependent appearance of MHC II+ and CD11c+ cell populations observed following mouse brain irradiation in our previous work [3] is dependent on CCL2/CCR2 signaling.

To address this hypothesis, bone marrow chimera animals were created using marrow from animals engineered to constitutively express enhanced green fluorescent protein (eGFP) under the β-actin promoter and, following confirmation of chimerism, the animals underwent brain irradiation. In support of our hypothesis, radiation exposure resulted in a chronic, dose-dependent increase in the number of eGFP-expressing cells in the brain. Furthermore, chimeras created with CCR2-deficient, eGFP+ bone marrow demonstrated reduced infiltration levels of eGFP+ myeloid cells following CNS radiation, indicating the importance of CCR2 signaling in myeloid cell recruitment in this injury model.

## Methods

### Animals

This study was carried out in strict accordance with the recommendations in the Guide for the Care and Use of Laboratory Animals of the National Institutes of Health. Animal protocols were reviewed and approved by the University of Rochester Institutional Animal Care and Use Committee (Protocol Number: 2004-74). Male C57BL/6J (stock no. 000664), C57BL/6-Tg(CAG-EGFP)10sb/J (stock no. 003291), and B6.129S4-*Ccr2*^*tm1Ifc*^/J (stock no. 004999) mice were purchased from The Jackson Laboratory at 8 to 10 weeks of age. Animals were housed 5 to a cage in temperature (23 ± 3^o^C) and light (12:12 light/dark) controlled rooms with free access to chow and water. Mice were routinely monitored for health issues and had no observable problems at the time of euthanasia.

### Bone marrow chimeras

C57BL/6J mice were exposed to two 6 Gy doses of whole body irradiation, separated by 4 h, using a ^137^Cs source (J. L. Shepherd and Assoc.) at a dose rate of 1.9 Gy/min. The mice were unanesthetized, confined in customized plexiglass jigs, and placed on a slit collimator such that the radiation field included the entire mouse, with the head shielded in order to reduce the dose to the head to 15–20 % of the unshielded area. Head shielding was used in order to minimize the split dose effect with respect to the subsequent brain exposures since we observed significant increases in the number of eGFP+ cells in the CNS of non-shielded animals compared to head-shielded animals following brain irradiation (Additional file 1: Figure S1). Indeed, other investigators also have found increased immune cell infiltration in bone marrow transplanted animals where the cranium was exposed to radiation as part of the transplantation process [26, 45]. Following the second dose of total body radiation, the mice were injected intravenously with 4 × 10^6^ bone marrow cells harvested from the femurs and tibias of mice that constitutively express enhanced green fluorescent protein (eGFP) and then returned to the vivarium for 6 weeks, allowing for bone marrow reconstitution. To determine the role of CCR2 signaling, we used an identical procedure to create chimeric C57BL/6J mice reconstituted with bone marrow from eGFP-expressing mice that had been crossed with mice deficient for CCR2. In all studies, the degree of bone marrow reconstitution was confirmed by drawing peripheral blood prior to euthanasia. Flow cytometry was subsequently performed to confirm the percentages of eGFP+ cells expressing CD11b, B220, CD4, and CD8, representing monocytic, B cell, and T cell lineages, respectively.

### Brain irradiation

For brain irradiation, mice were anesthetized (ketamine [90 mg/kg] and xylazine [8 mg/kg], administered intraperitoneally) then laid supine on the ^137^Cs irradiator such that the brain volume between their eyes and ears was exposed using a 5-mm × 12.2-cm collimator slit. This collimator provided a uniform field at a dose rate of 1.25 Gy/min with sharp edges that fell to a dose rate of 0 Gy/min within 2.5 mm of the slit edge. Animals (*n* = 6 per group) were exposed to a dose range of 0 to 45 Gy. After irradiation, mice were returned to the vivarium and supplied with laboratory diet and water ad libitum until the time of euthanasia. Mice in the time course study (35 Gy only) were euthanized at 1 day, 3 days, 7 days, 1 month, 3 months, or 6 months post-radiation; mice in the dose range and CCR2 chimera studies were euthanized at 6 months post-radiation.

### Tissue collection

Immediately after euthanasia, mice were flushed intracardially with 10–15 ml of a solution containing 2 IU/ml heparin and 0.05 % sodium nitrite in 0.15 M phosphate buffer (PB) and then perfused with 50 ml of 4 °C 4 % paraformaldehyde dissolved in 0.15 M PB (pH = 7.2). Brains were removed and post-fixed in 4 % paraformaldehyde dissolved in 0.15 M PB (pH = 7.2) at 4 °C for 2 hours protected from light. Brains were then transferred to a 30 % sucrose solution in 0.15 *M* PB at 4 °C, protected from light until equilibrated, and were then snap frozen in isopentane and stored at −80 °C until sectioning.

### Immunohistochemistry/immunofluorescence assays

Brains were sectioned in the coronal plane at 30 μm on a sliding knife microtome with a −25 °C freezing stage. Sections were stored in cryoprotectant at −20 °C until further processing. Visualization of antibody-bound sections for immunohistochemistry (IHC) was performed using biotinylated secondary antibodies, avidin-biotin complex (Elite), and 3,3-diaminobenzadine (DAB) substrate kit (Vector Laboratories). The following primary antibodies were utilized: mouse MHC class II I-A^b^ (BD Pharmingen, 1:2000); Iba-1 (Wako, 1:5000); ICAM-1 (AbD Serotec, 1:2000); rat anti-CD3 (AbD Serotec, 1:4000); hamster anti-CD3 (Santa Cruz Biotechnology Inc., 1:2000); CD11c (BD Pharmingen, 1:500); and CD11b (Invitrogen, 1:1000). Biotinylated secondary antibodies utilized included goat anti-Armenian hamster IgG (Jackson Laboratories, 1:1000); goat anti-mouse F(ab′)2 (Jackson Laboratories, 1:2000); goat anti-rabbit IgG (Vector Laboratories, 1:1000); and goat anti-rat IgG (Vector Laboratories, 1:2000). Sections used for MHC II, CD11c, CD3, and Iba-1 analysis were counterstained with Methyl Green (Vector Laboratories) according to the manufacturer’s protocol. For immunofluorescence, primary antibodies were utilized at concentrations three times the IHC level. Secondary antibodies bound to Alexa (Invitrogen) or DyLight (Jackson Laboratory) fluorophores were utilized at a dilution of 1:500.

### Cell quantification

Profiles of eGFP+ cells were quantified in three distinct brain regions: the fimbria/fornix, corpus callosum/extreme capsule, and hippocampus, regions that were completely within the radiation field. Counting for this analysis began rostrally with the first section where the dentate gyrus was visible and ceased caudally with the last section containing the hippocampus. For the construction of the graph demonstrating the distribution of eGFP+ cells along the rostro-caudal axis (Fig. 3), eGFP+ cell profiles were counted on sections throughout the brain until the end of the cerebral cortex for each animal. In both of the aforementioned cases, bright-field images of the sections were captured using a RT Spot camera (Diagnostic Instruments, Inc.) using a ×10 objective and were combined using the photomerge function in Adobe Photoshop CSII (Adobe Systems). Total areas for the regions examined were calculated using ImageJ (NIH), and the total number of cell profiles was divided by the total area in square millimeters. In the case of Fig. 3, the number of cells per square millimeter was plotted versus the approximate distance to/from bregma, which was estimated using a mouse brain atlas [46].

To quantify MHC II+, CD11c+, and CD3+ cells, sections were viewed with a Zeiss Axioplan2i light microscope (Zeiss, Thornwood, NY, USA) and the numbers of positively stained cells with a methyl green-stained nucleus were derived from the total seen in the first two brain sections containing the dentate gyrus. The total number of MHC II+, CD11c+, and CD3+ stained cell profiles co-localizing with a methyl green positive nucleus were also plotted for each time point. A two-way ANOVA was conducted on the values associated with each marker from both chimera animals and non-chimera animals at 6 months post-brain irradiation. When the interaction term of the two-way ANOVA was significant, Fisher’s protected least significant differences post hoc tests were conducted comparing all groups.

To quantify Iba-1+ cells in the hippocampus, sections were viewed with a Zeiss Axioplan light microscope equipped with a Prior motorized stage. Morphometric data were collected using the stereological program Stereologer (Systems Planning and Analysis, Inc.) and its optical disector method for unbiased cell counting. The number of cells was reported as the total number of Iba-1-positive hippocampal cells. Data were compared by an unpaired *t* test using Prism 5.01 (GraphPad Software, www.graphpad.com). A *p* value <0.05 was considered to be statistically significant.

## Results

### Chimera creation did not affect the peripheral or CNS response to brain irradiation

In order to determine any effect of, or interaction between, chimera induction and subsequent brain irradiation on circulating BMD cell populations, peripheral blood was collected at all time points and analyzed using FACS. The percentage of cells expressing CD11b, B220, CD4, or CD8 (representing monocytic, B cell, and T cell lineages, respectively) and also expressing eGFP were calculated and were compared for each cell marker using a two-way ANOVA with Bonferroni post hoc tests. No significant difference in the interexperimental degree of chimerism (relative levels of bone marrow reconstitution) was seen between chimera groups alone or, importantly, between brain-irradiated versus non-brain-irradiated chimeras at any of the different time points for the majority of cell types (data not shown). The only exception was the CD4+ population at 3 days post-brain irradiation, when a significant, but transient, decrease in the percentage of eGFP+ cells was seen (*p* < 0.01). The transience of this response, likely an indicator of the known radiation sensitivity of CD4+ cells, was felt to be insufficient to affect the responses seen at the remaining time points.

**Fig. 1:**
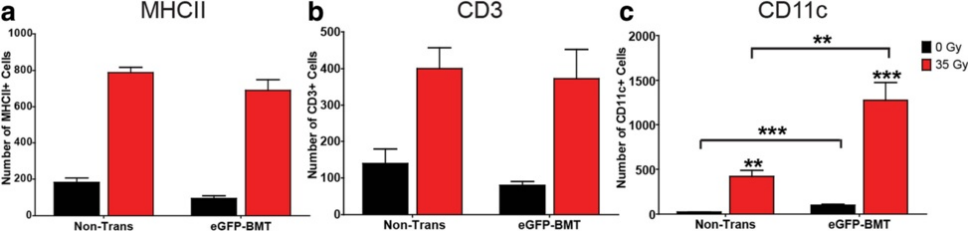
IHC analysis of immune cell markers in eGFP chimeras at 6 months following brain irradiation. To determine if the radiation component of chimera induction altered the presence of immune markers seen following brain irradiation in wild-type animals, 0- and 35-Gy brain-irradiated eGFP chimera (bone marrow transplant: eGFP-BMT) and non-chimera (Non-Trans) animals were analyzed for **a** MHCII+, **b** CD3+, and **c** CD11c+ cells at 6 months post-irradiation. The numbers of positively stained cells quantified in the first two sections containing the dentate gyrus as described in the Methods section were compared using a two-way ANOVA with Fisher’s protected least significant (PLSD) post hoc tests comparing all conditions. *Graph bars* represent mean ± SEM, *n* = 4–6 mice per condition: **p ≤ 0.01 and ***p ≤ 0.001

In addition to assessing the peripheral/humoral response to chimera induction, IHC staining was performed to determine whether the inflammatory responses seen in the brains of chimeras were similar to that seen in wild-type, non-chimera animals. Staining for MHCII, CD3, and CD11c was conducted on control and 35-Gy-irradiated tissues at 6 months post-bone marrow transplant (Fig. 1). The numbers of positively stained cells for each marker in transplanted wild-type animals were compared to the numbers attained from non-transplanted wild-type animals using a two-way ANOVA. If the interaction term was significant, it was followed by Fisher’s PLSD post hoc tests.

For both MHC II+ and CD3+ cells, the two-way ANOVA revealed no significant interaction between brain irradiation and bone marrow transplant/chimera induction (Fig. 1a, b), although a significant effect of bone marrow transplantation on MHC II+ cells (*F*(1, 16) = 6.429, *p* = 0.0220) was found (Fig. 1a). Both MHC II+ and CD3+ cells demonstrated a significant effect of brain irradiation (*F*(1, 16) = 263.026, *p* < 0.0001; *F*(1, 17) = 26.45, *p* < 0.0001, respectively), but the fact that there was no significant difference between the number of MHC II+ or CD3+ cells in animals undergoing chimera induction followed by brain irradiation versus those undergoing brain irradiation alone suggests little to no additive effect from chimera induction.

Statistical analysis of CD11c+ cell numbers revealed a significant effect from the chimera induction/BMT protocol (*F*(1, 16) = 13.42, *p* = 0.002), a significant effect of brain radiation exposure (*F*(1, 16) = 37.99, *p* < 0.0001), and also a significant interaction effect (*F*(1, 16) = 9.369, *p* = 0.0075) (Fig. 1c). However, since the number of CD11c+ cells was significantly different between the chimera/BMT and non-chimera control groups, the fold change in CD11c+ cells was calculated for irradiated mice with respect to their controls for both conditions to determine whether the CD11c response to brain irradiation differed between the two sets of animals. An unpaired *t* test found that the fold change in the number of CD11c+ cells following brain irradiation between non-chimera (*M* = 17.7, SD = 2.87) and chimera animals (*M* = 12.69), *t*(9) = 1.469, *p* = 0.1760) did not reach statistical significance, supporting our contention that the irradiation component of chimera induction did not affect the overall cell response to brain irradiation.

### Brain irradiation induced dose-dependent, delayed infiltration of bone marrow-derived cells

**Fig. 2:**
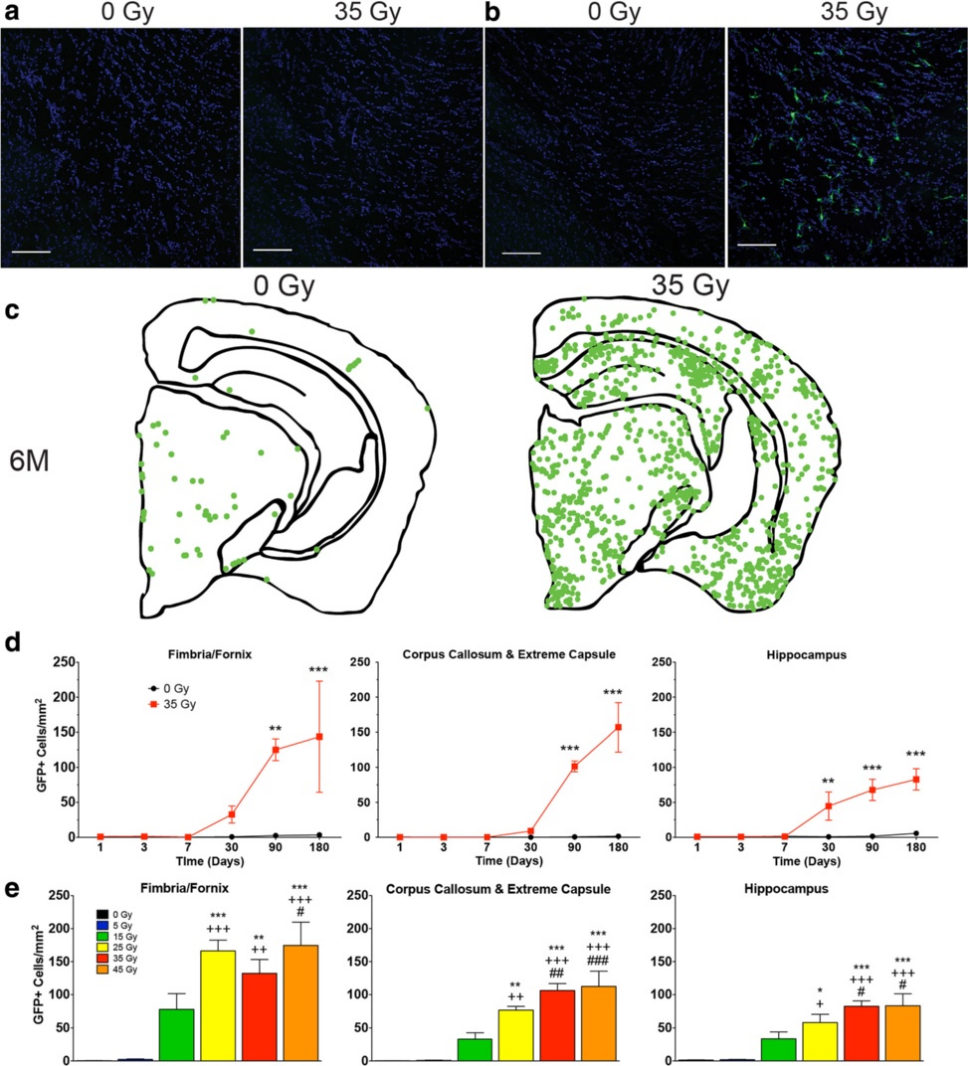
Brain irradiation induced delayed, dose-dependent infiltration of bone marrow-derived cells. Chimera mice with bone marrow-expressing eGFP received brain irradiation (0 or 35 Gy) and were sacrificed at multiple time points. Sections from irradiated mice at **a** 1 day and **b** 6 months post-irradiation were stained with Hoechst solution, and photomicrographs were taken of the internal capsule. *Scale bars*: 100 μm. **c** Images illustrating the distribution of eGFP+ cells at 6 months were constructed for 0 and 35 Gy animals. Each *dot* represents one eGFP-expressing cell. **d** eGFP+ cells per square millimeter were quantified in three brain regions (the fimbria/fornix, corpus callosum/extreme capsule, and the hippocampus), beginning with the first section caudal to bregma containing fimbria or fornix. Data were analyzed by two-way ANOVA and Bonferroni post hoc tests comparing irradiated animals to controls for each time point, *n* = 4–5 mice per condition: **p ≤ 0.01 and ***p ≤ 0.001. **e** Chimera animals were irradiated with 0, 15, 25, 35, or 45 Gy to brain, sacrificed at 6 months post-irradiation and analyzed for eGFP+ cells per square millimeter. Data were analyzed by one-way ANOVA and Bonferroni post hoc tests comparing all of the doses, *n* = 4–5 mice per condition. Symbols used to distinguish statistical significances between doses: *0 Gy, *+* 15 Gy, and ^#^25 Gy. One symbol represents *p* ≤ 0.05, two symbols represent *p* ≤ 0.01, and three symbols represent *p* ≤ 0.001

To provide a detailed characterization of the effect of CNS irradiation on the observed recruitment of peripherally derived immune cells to the brain, chimeras were administered brain irradiation (0 or 35 Gy) and sacrificed at multiple time points. Initial observation of tissues showed no evidence of eGPF+ cell infiltration at early time points, but clear evidence of infiltration at later time points (Fig. 2a, b). Images were constructed to illustrate the distribution of recruited cells at these later time points (Fig. 2c), similar to our previously reported findings [3]. Based on these findings, the number of eGFP+ cells per square millimeter was quantified across all time points for three distinct brain regions, two white matter and one gray matter: (1) the fimbria/fornix, (2) corpus callosum and extreme capsule, (3) and hippocampus, respectively (Fig. 2d). At 1 month post-radiation, an increase in the numbers of cells per square millimeter was seen in all brain regions, although statistical significance was only achieved in the hippocampal region (45 ± 40 vs. 1 ± 1, *p* < 0.01). However, by 3 months post-radiation, a statistically significant increase in the number of eGFP+ cells per square millimeter was seen in all brain regions, with the density increasing further at 6 months (Fig. 2d). The data were analyzed using two-way ANOVA with Bonferroni post hoc tests comparing brain-irradiated chimeras to controls at each time point. Significant effects of brain radiation exposure (*p* = 0.001, *p* < 0.0001, and *p* < 0.0001), effects of time (age post-radiation) (*p* = 0.005, *p* < 0.0001, and *p* < 0.0001), and effects of interaction between brain irradiation and time (*p* = 0.007, *p* < 0.0001, and *p* < 0.0001) were found in the fimbria/fornix, corpus callosum/extreme capsule, and hippocampal regions, respectively. Interestingly, the majority of eGFP+ cells observed exhibited microglial morphology, and the infiltration pattern showed a rostro-caudal distribution, with little evidence of infiltration in the extreme rostral or caudal sections.

To determine the effect of dose on infiltration, the study was repeated using a range of brain irradiation doses (5–45 Gy). At 6 months post-radiation, the number of eGFP+ cell per square millimeter was again quantified for the three brain regions and analyzed using one-way ANOVA with Bonferroni post hoc tests, and comparisons were performed between all doses (Fig. 2e). Significant differences in the number of infiltrating cells were found with respect to radiation dose in the fimbria/fornix region (*F*(5, 24) = 14.77, *p* < 0.0001), the corpus callosum/extreme capsule region (*F*(5, 24) = 20.16, *p* < 0.0001), and the hippocampus (*F*(5, 24) = 13.22, *p* < 0.0001). Post hoc test analysis revealed that brain irradiation resulted in dose-dependent increases in cell infiltration in all brain regions, which reached significance ≥25 Gy, although infiltration was clearly evident at 15 Gy (Fig. 2e).

### Direct radiation exposure is required for peripherally derived immune cell recruitment into CNS

**Fig. 3:**
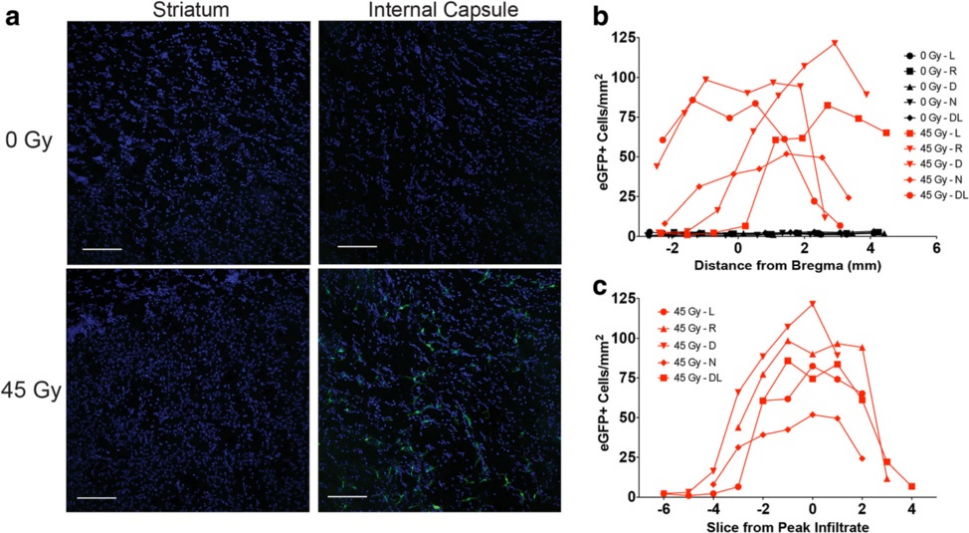
Distribution of eGFP+ cells along the rostro-caudal axis. Chimera mice with bone marrow constitutively expressing eGFP received brain irradiation (0 or 45 Gy) and were sacrificed at 6 months post-radiation. **a** Sections taken 1:24 were stained with Hoechst solution, and photomicrographs of the striatum and internal capsule for both control and irradiated mice were taken. *Scale bars*: 100 μm. **b** The number of eGFP+ cells per square millimeter was plotted in relation to the estimated distance from bregma (mm) in 0- and 45-Gy brain-irradiated animals. **c** The number of eGFP+ cells per square millimeter in 45-Gy brain-irradiated chimera mice was plotted in relation to the section containing the peak infiltrate of eGFP+ cells

As is often recognized clinically, the radiation-induced injury seen in this study, defined by upregulation of activation and immune markers, areas of increased staining and altered morphology, was limited to the tissue volume directly exposed to the radiation beam. This pattern of response appeared to be repeated with respect to inflammatory cell infiltration since the rostral striatum, which was shielded by the irradiator collimator, failed to demonstrate cellular infiltration at any dose compared to the internal capsule, which was within the radiation beam (Fig. 3a). To better establish whether the directly injured tissue selectively influenced the influx of peripherally derived immune cells, a profile of eGFP+ cells was established from a 1:24 series of brain tissue sections from 0- or 45-Gy-irradiated animals. The number of cells per square millimeter was plotted versus the location relative to bregma of the quantified tissue section (Fig. 3b); locations were estimated using a mouse brain atlas [46]. In the control (non-brain irradiated) chimera animals, the number of cells per square millimeter did not change significantly between tissue sections along the rostro-caudal axis, regardless of position in relation to bregma. Furthermore, the quantification of tissue sections from non-irradiated chimeras indicated only ≤3 eGFP+ cells per square millimeter. In contrast, sections from 45-Gy brain-irradiated chimera animals exhibited a greater range of values (1–121 cells/mm^2^), with every animal assessed exhibiting a similar pattern regarding cell number change (increase, plateau, then decrease) along the rostro-caudal axis. Although the position of the peak response varied along the axis, the distance over which the change in cell numbers occurred was observed as being relatively similar (within approximately 7–8 brain sections). To confirm this observation, Fig. 3b was re-plotted, using the tissue section that showed the greatest infiltrate per square millimeter as section 0 (Fig. 3c). Since the sections were cut at 30-μm intervals and mounted 1:24, multiplying the number of sections over which the number of eGFP+ cells was greater than that of controls yielded a radiation-responsive distance of 5.04–5.76 mm, relatively close to the anticipated volume of radiation injury from a 5-mm slit, the exact size of the collimator utilized in our model, plus the additional injury due to Bremsstrahlung radiation.

### Bone marrow-derived cells express CD11c, MHC II, CD11b, and CD3 in irradiated brain

**Fig. 4:**
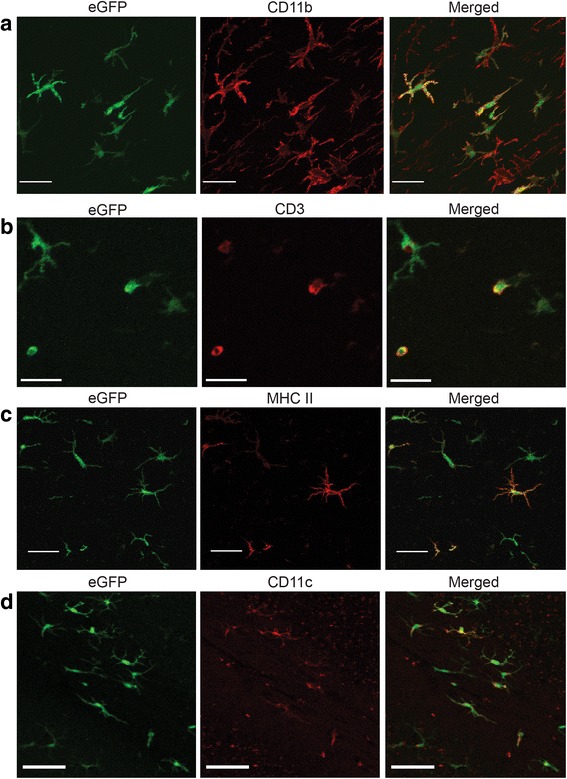
Characterization of infiltrating immune cells. Sections from 35 Gy brain-irradiated, eGFP+ chimera animals at 6 months post-radiation were stained for **a** CD11b, **b** CD3, **c** MHC II, or **d** CD11c. *Scale bars*: **a**, **b** 20 μm, **c** 30 μm, and **d** 50 μm

**Fig. 5:**
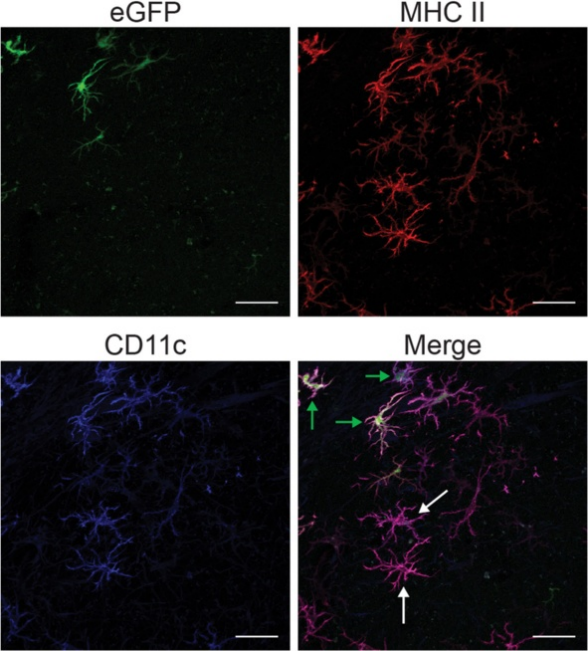
Brain irradiation resulted in the recruitment of CD11c+/MHC II+ cells. Sections from 35 Gy brain-irradiated eGFP+ chimera animals at 6 months post-radiation were stained for MHC II and CD11c. Co-localization of markers were seen in eGFP-negative, endogenous cells (*white arrows*) as well as eGFP+ peripherally derived cells (*green arrows*). *Scale bars*: 100 μm

To further characterize radiation-induced myeloid cell recruitment and determine their contribution to the observed neuroinflammatory response, sections from chimera animals exposed to 0 versus 35 Gy brain irradiation at six months post-radiation were stained for eGFP and the immune marker, CD11b. Visual examination revealed that a large proportion of the eGFP+ cells co-labeled with CD11b, indicating recruitment of cells of myeloid origin (Fig. 4a), although independent staining for MHC II, CD3, and CD11c confirmed the recruitment of infiltrating cells of multiple phenotypes into the brain parenchyma (Fig. 4b–d), as was reported in our previous study [3]. To determine any interaction between these various cell populations, additional co-localization studies were performed (Fig. 5). Cells co-expressing eGFP with MHC II and CD11c were seen adjacent to cells expressing MHC II and CD11c only, suggesting that the inflammatory population was comprised of both endogenous and peripheral cells.

**Fig. 6:**
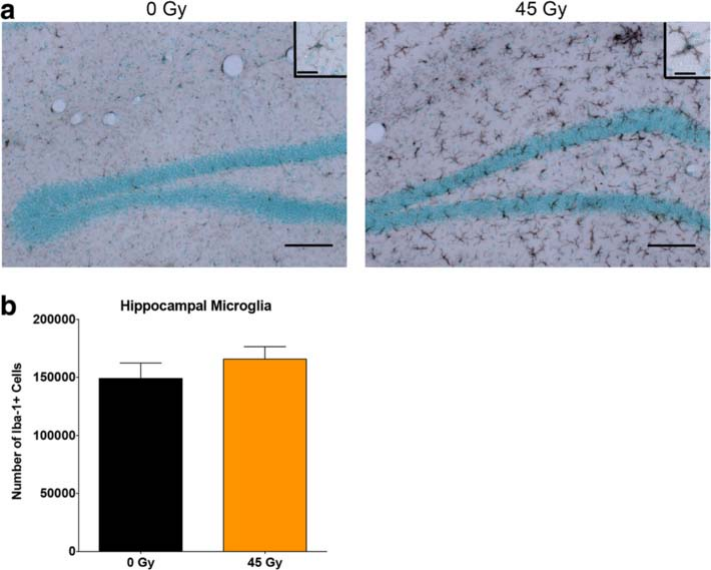
Stereologic quantification of hippocampal myeloid cells in 0 and 45 Gy brain-irradiated, eGFP+ chimera mice at 6 months post-irradiation. **a** Sections from eGFP chimeras at 6 months following 0 or 45 Gy brain radiation were stained for Iba-1 and counterstained for methyl green (Vector Laboratories). *Scale bars*: 100 μm; *inset*, 20 μm. **b** Graphical representation of the total number of Iba-1+ cells in the hippocampi of 0 and 45 Gy brain-irradiated chimeras. The average numbers of cells were compared using a two-tailed *t* test. *Graph bars* represent mean ± SEM, *n* = 6 mice per condition

Based on the cell profile density, the average number of infiltrating cells in the hippocampus at 6 months post-radiation was calculated to be ~34.3 × 10^3^ cells. To determine if this recruitment resulted in an increase in the total number of myeloid cells, sections from 0 and 45 Gy exposed animals at 6 months post-radiation were stained for Iba-1 and the number of Iba-1+ cells in the hippocampus was quantified using unbiased stereology. Although there was a clear difference in morphology and staining intensity between control and irradiated tissues (Fig. 6a), an unpaired *t* test revealed no significant difference in the total number of microglial/myeloid cells between brain-irradiated animals and controls in the hippocampus at this time point (Fig. 6b), suggesting that the infiltrating cells had replaced, but not added to, the population of microglial resident cells.

### Radiation-induced recruitment of peripherally derived myeloid cells is dependent on CCR2 signaling

**Fig. 7:**
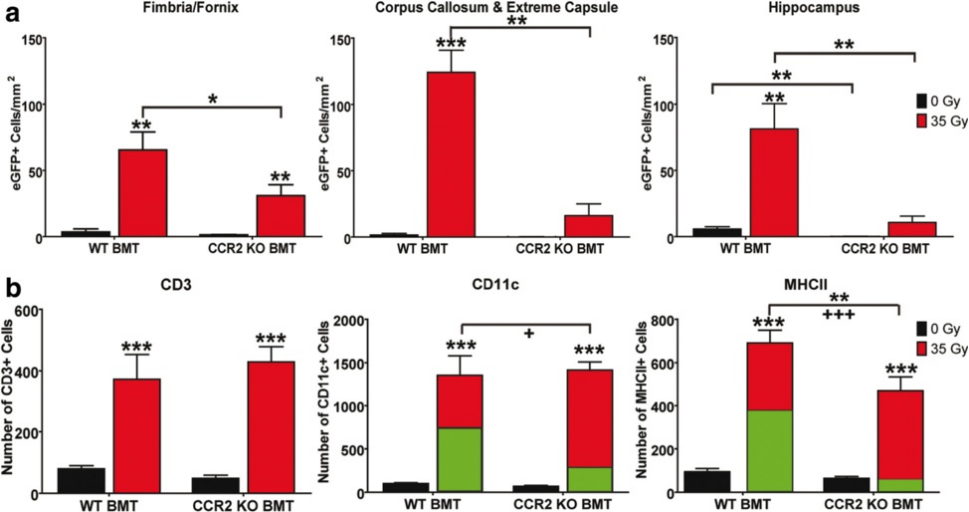
Effect of CCR2 signaling on immune cell infiltration following brain irradiation. **a** eGFP+ CCR2-null chimeras were exposed to 0 or 35 Gy brain irradiation and sacrificed at 6 months post-radiation. The numbers of eGFP+ cells per square millimeter were calculated for three regions of interest (the fimbria/fornix, corpus callosum/extreme capsule, and hippocampus). Data were compared to eGFP+ CCR2-competent chimeras using a two-way ANOVA and Bonferroni post hoc tests. **b** Sections from eGFP+ CCR2-null chimeras versus eGFP+ CCR2-competent animals were stained immunofluorescently for CD3, MHC II, or CD11c. The number of positively stained cells also expressing eGFP was calculated for both the MHC II and CD11c analyses: total numbers of CD11c+ or MHC II+ cells are represented by the *red bars*, cells co-localizing with eGFP are represented by the *green portion* of the bars. All *graph bars* represent means ± SEM, *n* = 4–6 mice per condition: *p ≤ 0.05, **p ≤ 0.01, and ***p ≤ 0.001. For the percent of positively stained cells that also expressed eGFP, +*p* ≤ 0.05, +++*p* ≤ 0.001

As noted, we had observed that the majority of infiltrating cells following brain irradiation appeared to display a microglia- or myeloid-like morphology (Figs. 5 and 6). CCR2 is a chemokine receptor recognized as playing a critical role in the recruitment of monocyte cells in various disease models [47]. Previous work from our laboratory demonstrated early and late increases for CCR2’s primary ligand, CCL2 (MCP-1) [1, 3]. To examine the role of CCL2/CCR2 signaling in immune cell recruitment seen following brain irradiation, animals deficient for CCR2, but constitutively expressing eGFP under the β-actin promoter, served as donors for chimera studies. Following confirmation of chimerism, animals were exposed to 0 or 35 Gy brain irradiation and sacrificed at 6 months post-irradiation. The numbers of eGFP+ cells in the eGFP+ CCR2-null chimeras were quantified in the three regions of interest in the brain and compared to eGFP+ CCR2+ animals utilizing two-way ANOVAs, followed by Fisher’s PLSD post hoc tests comparing all groups (Fig. 7a). Importantly, transplant efficiencies analyzed by two-tailed, unpaired *t* test for the eGFP+ CCR2-null versus eGFP+ CCR2+ chimera animals showed no significant difference in the percentage of eGFP+ CD45+ cells in peripheral blood between the two sets of transplanted animals.

Analysis of area-specific infiltrating (eGFP+) cells in the eGFP+, CCR2-null chimeras at 6 months post-radiation (Fig. 7a) showed a significant interaction effect (*F*(1, 17) = 5.15, *p* = 0.04; *F*(1, 17) = 43.69, *p* < 0.0001; *F*(1, 17) = 17.24, *p* = 0.0007) and a significant effect of brain irradiation *F*(1, 17) = 41.56, *p* < 0.0001; *F*(1, 17) = 73.83, *p* < 0.0001; *F*(1, 17) = 30.30, *p* < 0.0001) in the fimbria/fornix, corpus callosum and extreme capsule, and hippocampus, respectively, as would be anticipated, but also demonstrated a significant effect of bone marrow genotype (*F*(1, 17) = 6.61, *p* = 0.02; *F*(1, 17) = 46.08, *p* < 0.0001; *F*(1, 17) = 23.70, *p* = 0.0001). No significant difference in the number of eGFP+ cells per square millimeter was found between CCR2+ and CCR2-null 0 Gy control animals in any of the regions of interest, indicating that there was no baseline effect on recruitment. However, post hoc test analysis showed a significant difference in the number of eGFP+ cells per square millimeter between genotypes in all areas of interest (*p* = 0.05, 0.003, and 0.002 for the fimbria/fornix, corpus callosum and extreme capsule, and hippocampus, respectively) following irradiation, with a significant decrease in the number of eGFP+ cells seen in all areas of interest in the CCR2-null chimeras, suggesting that CCR2 signaling does indeed play a role in radiation-induced cellular recruitment to the CNS (Fig. 7a).

To investigate whether CCR2 deficiency had a differential effect on the radiation-induced recruitment of the specific immune cell types in the brain, tissue sections from irradiated and control eGFP+ CCR2-null chimeras were stained for CD3, CD11c, and MHC II, quantified and compared to eGFP+ CCR2+ animals. Data were analyzed by two-way ANOVA, followed by Fisher’s PLSD post hoc tests comparing control and brain-irradiated animals at 6 months post-radiation (Fig. 7b). Analysis of the CD3+ cells demonstrated a significant effect of radiation exposure (*F*(1, 15) = 49.93, *p* < 0.0001), but no significant effect with respect to marrow genotype, suggesting that radiation-induced T cell infiltration was independent of CCR2 signaling. Analysis of the number of CD11c+ cells also showed a significant effect of brain irradiation (*F*(1, 15) = 109.0, *p* < 0.0001) but no significant interaction effect or effect of marrow genotype. However, using an unpaired, two-tailed *t* test with Welch’s correction to compare the number of CD11c+ (resident) cells to CD11c+ cells showing co-localization with eGFP+ (BMD/recruited) revealed a significant reduction in the number of recruited CD11c+ cells in the eGFP+ CCR2-null chimeras (*M* = 284.0, SD = 48.6) compared to the eGFP+ CCR2+ chimeras (*M* = 773.5, SD = 164.4) (*t*(4) = 2.856, *p* = 0.0461) despite the overall total numbers of CD11c+ cells remaining the same. These findings strongly suggest that CCR2 signaling plays a role in the recruitment of myeloid CD11c+ cells to the brain following irradiation, but that, in the absence of CCR2 signaling, compensatory mechanisms allow for amplification of the resident microglial (CD11c+) population.

Analysis of the MHC II+ cells suggest that a slightly different CCR2-related response was elicited in this cell population, with a significant effect now being seen on total cell numbers with respect to marrow genotype (*F*(1, 17) = 8.59, *p* = 0.009). Post hoc testing, using Fisher’s PLSD, showed a significant decrease in the number of MHC II+ cells following brain irradiation in the CCR2-null chimeras (*p* = 0.0023) (Fig. 7b). In addition, further analysis by unpaired, two-tailed *t* test with Welch’s correction again revealed a decrease in the number of MHC II+, eGFP+ cells in the eGFP+ CCR2-null chimeras (*M* = 57.9, SD = 18.9) relative to the eGFP+ CCR2+ animals (*M* = 381.8, SD = 40.9) (*t*(5) = 7.190, *p* = 0.0008). As had been seen with respect to the CD11c+ cells, these findings suggest that CCR2 signaling also plays a role in the recruitment of MHC II+ cells to the brain following irradiation, but that compensatory mechanisms allowing for amplification of the resident MHC II+ population are less robust in the absence of CCR2 signaling.

## Discussion

Earlier work from our group [3] and others [17, 31] has demonstrated that brain irradiation leads to delayed influx of peripherally derived leukocytes in the CNS that becomes apparent as early as 7 days post-irradiation. Moreover, we demonstrated the persistence of peripherally derived cells in the parenchyma out to 1 year post-radiation [3]. In combination with the increased MHC II expression noted in our previous experiment, these findings led us to postulate that increased MHC II expression may result from radiation-dependent recruitment of peripherally derived myeloid cells as reported by Burrell et al. [31] and that infiltration of these cells is dependent on CCR2 signaling, which has been shown to be important for myeloid cell recruitment in other models of CNS injury [47].

The series of studies presented utilized bone marrow chimeras. Use of this model may be felt by some to confound data interpretation since irradiation is part of the induction process, adding an additional earlier radiation injury; however, our use of head shielding during the creation of chimeras appeared to effectively abrogate the cell infiltration seen in chimeras created without head shielding (Additional file 1: Figure S1). Moreover, the few eGFP+ cells observed in our control chimeric mice (0.5–3 cells/mm^2^) were nearly all associated with the vasculature, which contrasts with the parenchymal location of infiltrating cells observed following brain irradiation. It also is possible that some of the differences seen in the various cell populations may have been the result of the transplantation process itself. However, there were no significant differences between the infiltrative response of the various cell types to brain irradiation between chimera and non-chimera animals, with the sole exception of the CD11c+ population. With respect to that population, chimeric animals were found to have increased numbers of CD11c+ cells at baseline compared to non-transplanted animals (Fig. 1c), supporting the observation of a radiation-induced recruitment of CD11c+ cells from the periphery made by Simard and Rivest [17], which increased over time. In addition, the number of CD11c+ cells in the CNS has been shown to increase with normal aging [30]. Although chimera animals in our study were typically 6 weeks older than non-chimera mice (because of the time required for bone marrow reconstitution), this relatively small difference in their ages is unlikely to account for the difference observed in CD11c+ cell density 6 months after irradiation. Importantly, all remaining comparisons were made between chimera groups.

Our studies demonstrated that radiation exposure of the brain yielded a delayed dose-dependent infiltration of peripherally derived cells that began at 1 month post-irradiation and continued to 6 months (Fig. 2). The increased numbers of peripheral cells accumulating in the CNS with dose and time could be due solely to recruitment or to a combination of recruitment and proliferation; future studies using proliferative markers could help distinguish between these possibilities. Our temporal findings are consistent with those of Burrell et al. [31], although we were not able to demonstrate a significant difference in cell number at 7 days. Infiltration occurred at a brain radiation dose of 15 Gy, but not 5 Gy, and is consistent with observations from other laboratories showing bone marrow-derived cell infiltration in the irradiated brain with 10 and 13 Gy [44, 48] as well as our own experiments in unshielded chimera mice, which showed infiltration with a cumulative dose of 12 Gy (Additional file 1: Figure S1). Such doses are well within the range used for normal tissue margins in stereotactic radiosurgery [49, 50], but whether CNS infiltration of peripheral cells occurs with fractionated radiotherapy has not been investigated. The majority of infiltrating cells presented with a microglial phenotype and stained positively for CD11b, a marker of myeloid cells (Fig. 4a), although positive staining for other immune cell markers was also found on peripherally derived eGFP+ cells (Fig. 4b–d). The relative expression of other inflammatory markers in our study was similar to that seen by others [31], with a majority of BMD cells recruited to the CNS co-localizing with the myeloid markers CD11b, MAC3, and Iba-1. Our study also demonstrated the presence of cells staining positive for CD11c and MHC II in the brains of chimera mice following irradiation (Fig. 5). The majority of these cells were eGFP+, indicating that they were BMD. Because chimerism was incomplete, the degree to which endogenous CNS microglia were also activated to express CD11c and MHC II could not be determined.

The temporal pattern of leukocyte infiltration (Fig. 2) is consistent with the pattern of T cell recruitment seen in our previous study [3] and is similar to the pattern observed by Burrell et al., although they detected increased numbers of BMD cells as early as 7 days post-irradiation [31], as had Morganti et al. [44]. Possible explanations for the temporal discrepancy in infiltration between those studies and the present study include the use of different mouse strains, radiation doses, methods of cell quantification, statistical analyses, and the interval between bone marrow transplantation and cranial irradiation. Nonetheless, our work provides additional evidence that a component of the brain’s response to radiation injury is an accumulation of myeloid cells from the periphery that persists for up to 6 months.

Our finding that CCR2-deficient chimeras demonstrated decreased BMD cell recruitment (Fig. 7) strongly suggests that radiation-induced neuroinflammation leads to myeloid cell recruitment via a CCL2/CCR2-dependent mechanism. These findings are consistent with the results in other models of CNS injury that have demonstrated the importance of CCL2/CCR2 signaling for monocyte/macrophage recruitment [47, 51]. However, it is possible that the decrease in CNS infiltrating cells observed in the CCR2-deficient chimeras was due to a reduction in circulating inflammatory monocytes, since, in addition to its established role in migration of hematopoietic stem cells into tissues [40], CCR2 signaling also plays a role in migration of these cells out of the bone marrow [52, 53]. Further experiments will need to be conducted to better address this issue in the context of radiation injury.

Another possible explanation for the observed increase in BMD cell recruitment is a lack of self-renewal by endogenous microglial progenitor cells following radiation-induced damage. Despite the infiltration of peripherally derived cells following brain irradiation in this study, we did not find a significant increase in the total number of microglia in the hippocampus (Fig. 6). This is in contrast to the findings of Okonogi et al. who demonstrated increased numbers of Iba-1+ cells in the brain stem, basal ganglia, and cerebral cortex at 3 and 8 weeks following 13 Gy of brain irradiation [48]. One explanation for discrepancies between the two studies is that different brain areas were analyzed; the variable myeloid response to radiation in different brain regions has been described recently in rats [54]. In addition, our analysis was limited to counts at 6 months post-radiation, so it is possible that there may have been earlier increases in cell numbers that were subsequently lost; the study by Okonogi et al. only followed the animals out to 8 weeks post-radiation [48]. Importantly, the overall density of BMD cells in irradiated brain reported by Okonogi is similar to the density of BMD cells we observed, and they represent a modest fraction of all Iba-1 labeled cells in histological sections [48]. These findings contrast with results reported by Burrell et al. who observed up to 50 % replacement of microglia by BMD cells in their study [31]. This discrepancy might relate to the detection method utilized (two-photon microscopy). Further studies utilizing flow cytometry could be employed to better quantify numbers of infiltrating cells as a function of dose, time after irradiation, and brain region.

## Conclusions

In summary, we have shown that peripherally derived immune cells participate in the CNS response to radiation, although their specific role is not clear. Certainly, ample evidence exists for BMD cells playing important roles in the repair of multiple forms of CNS injury, including neurodegenerative disease [55–61]. These peripherally derived cells may possess a more activated phenotype and/or act as better phagocytes than endogenous microglial cells in clearing debris or dysfunctional molecules [17, 55]. We also demonstrated that CCR2 signaling was required for the efficient recruitment of myeloid cells to the CNS after irradiation. Interestingly, CCR2 deficiency has been shown to prevent hippocampal-dependent spatial learning and memory impairment observed after cranial irradiation [62], raising the possibility that BMD infiltrating cells might contribute to CNS dysfunction. However, other mechanisms may underlie these deficits; for example, CCL2 deletion improves recovery of neurogenesis following irradiation, and this effect was not associated with dramatic cell infiltration at the times or conditions tested [63]. A better understanding of the roles of BMD infiltrating cells and the mechanisms behind their recruitment may provide a means to exploit this phenomenon therapeutically to prevent injury and treat associated CNS diseases.

## Additional file

Additional file 1: Figure S1. Effect of head shielding during bone marrow depletion on immune cell infiltration. In this bone marrow transplant experiment, one set of animals experienced total body exposure to two doses of 6 Gy, while the other set was irradiated with head shielding. Both sets of animals were injected intravenously with 4 × 10^6^ eGFP-expressing bone marrow cells via the tail vein. Six weeks were allowed for reconstitution, and then the animals were anesthetized intravenously with a ketamine/xylazine (90 mg/kg/8 mg/kg) mixture and brought down to the irradiator in a similar manner to those animals that were cranially irradiated (sham irradiated). Animals were returned to the vivarium and sacrificed at 6 months post-anesthetization. The numbers of eGFP+ cells per square millimeter were calculated for the fimbria/fornix, the corpus callosum/extreme capsule, and the hippocampus. The average numbers of cells for section were compared using two-tailed *t* tests. HS = Head Shielded; TBI = Total Body Irradiated. Graph bars represent means ± SEM, *n* = 6 mice per condition: **p* ≤ 0.05, ***p* ≤ 0.01, and ****p* ≤ 0.001. (TIFF 2826 kb)

## Abbreviations

B220: cluster of differentiation 45 receptor
BMD: bone marrow-derived
CCL2: chemokine (C-C motif) ligand-2 (monocyte chemotactic protein 1)
CCR2: C-C chemokine receptor type 2
CD11b: cluster of differentiation 11b (integrin, alpha M)
CD11c: cluster of differentiation 11c (integrin, alpha X)
CD3: cluster of differentiation 3 (T3 complex)
CD4: cluster of differentiation 4
CD8: cluster of differentiation 8
CNS: central nervous system
CX3CL1: chemokine (C-X3-C motif) ligand-1 (fractalkine)
eGFP: enhanced green fluorescent protein
F4/80: EGF-like module-containing mucin-like hormone receptor-like 1 (EMR1)
Gy: gray
Iba-1: ionized calcium binding adapter molecule 1
ICAM-1: intracellular adhesion molecule-1
MHC II: major histocompatibility complex, class II
PB: phosphate buffer
