# Supplementary figures and images for: Brain radiation injury leads to a dose- and time-dependent recruitment of peripheral myeloid cells that depends on CCR2 signaling
Source: J Neuroinflammation. 2016 Feb 3;13:30. doi: 10.1186/s12974-016-0496-8 (PMC4738790; doi:10.1186/s12974-016-0496-8)

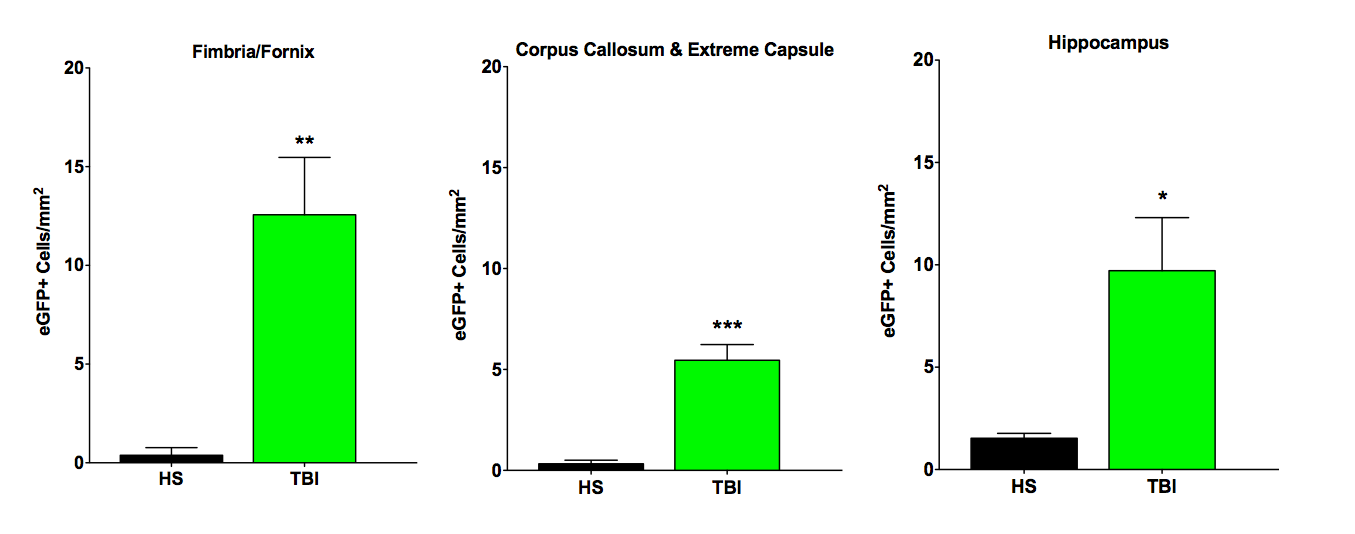

Supplement: Additional file 1: Figure S1. — Effect of head shielding during bone marrow depletion on immune cell infiltration. In this bone marrow transplant experiment, one set of animals experienced total body exposure to two doses of 6 Gy, while the other set was irradiated with head shielding. Both sets of animals were injected intravenously with 4 × 10^6^ eGFP-expressing bone marrow cells via the tail vein. Six weeks were allowed for reconstitution, and then the animals were anesthetized intravenously with a ketamine/xylazine (90 mg/kg/8 mg/kg) mixture and brought down to the irradiator in a similar manner to those animals that were cranially irradiated (sham irradiated). Animals were returned to the vivarium and sacrificed at 6 months post-anesthetization. The numbers of eGFP+ cells per square millimeter were calculated for the fimbria/fornix, the corpus callosum/extreme capsule, and the hippocampus. The average numbers of cells for section were compared using two-tailed t tests. HS = Head Shielded; TBI = Total Body Irradiated. Graph bars represent means ± SEM, n = 6 mice per condition: *p ≤ 0.05, **p ≤ 0.01, and ***p ≤ 0.001. (TIFF 2826 kb) [file 12974_2016_496_MOESM1_ESM.tiff]
